# Supplementary figures and images for: Role of Kindlin-2-Expressing Extracellular Vesicles in the Invasiveness of Triple Negative Breast Cancer Tumor Cells
Source: Cells. 2025 Jul 7;14(13):1034. doi: 10.3390/cells14131034 (PMC12248540; doi:10.3390/cells14131034)

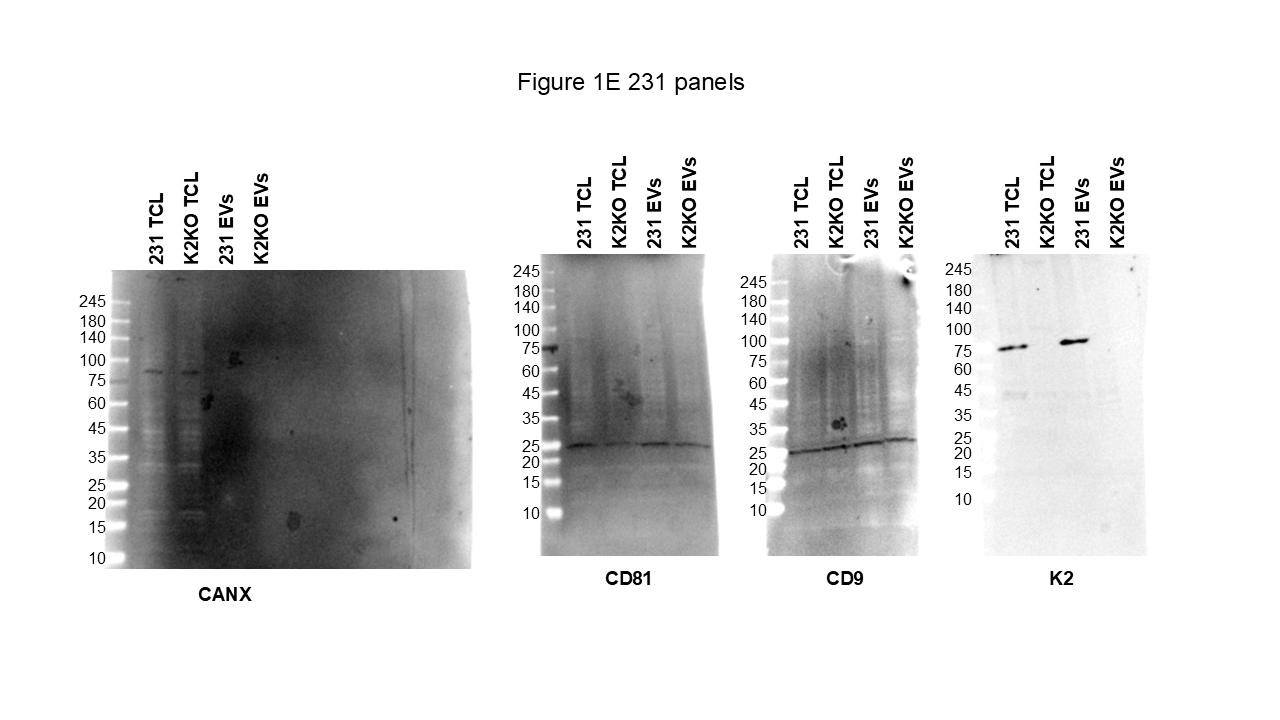

Supplement: Supplementary file 1 [file cells-14-01034-s001.zip › cells-3714860-supplementary/Uncropped WB/Uncropped WB for Figure 1D 231.tif]

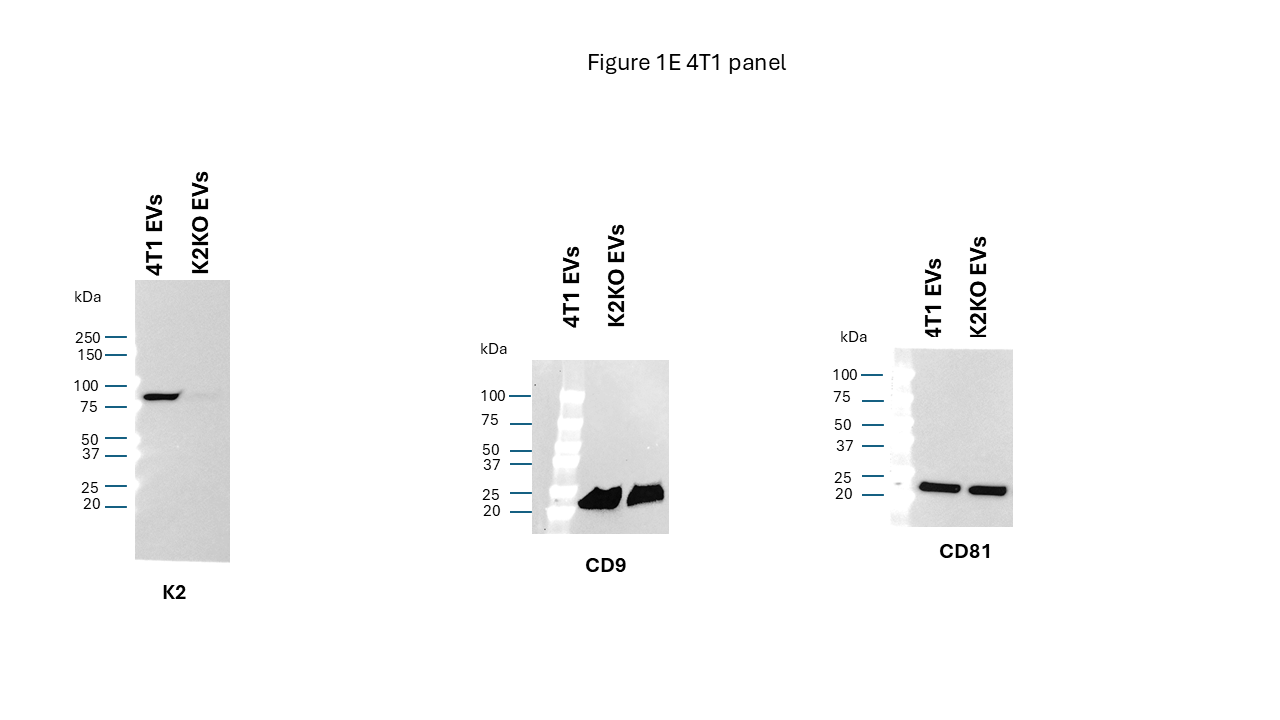

Supplement: Supplementary file 1 [file cells-14-01034-s001.zip › cells-3714860-supplementary/Uncropped WB/Uncropped WB for Figure 1D 4T1.TIF]

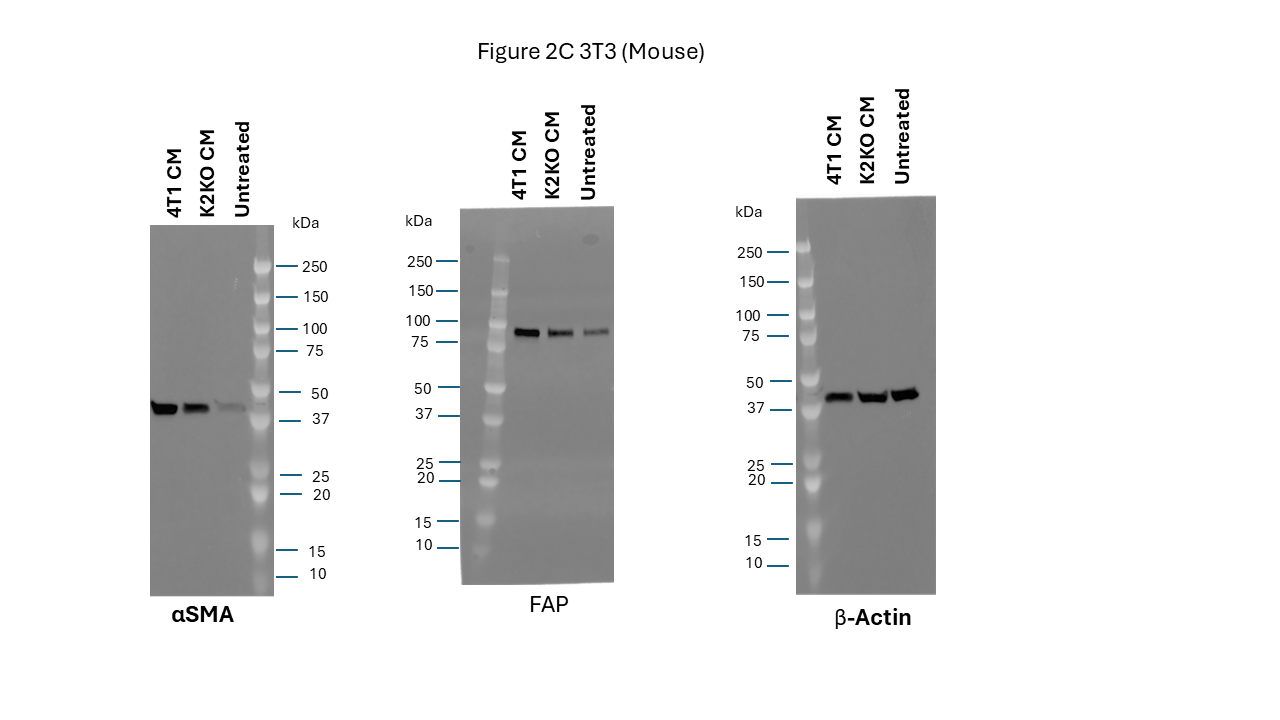

Supplement: Supplementary file 1 [file cells-14-01034-s001.zip › cells-3714860-supplementary/Uncropped WB/Uncropped WB for Figure 2C 3T3.TIF]

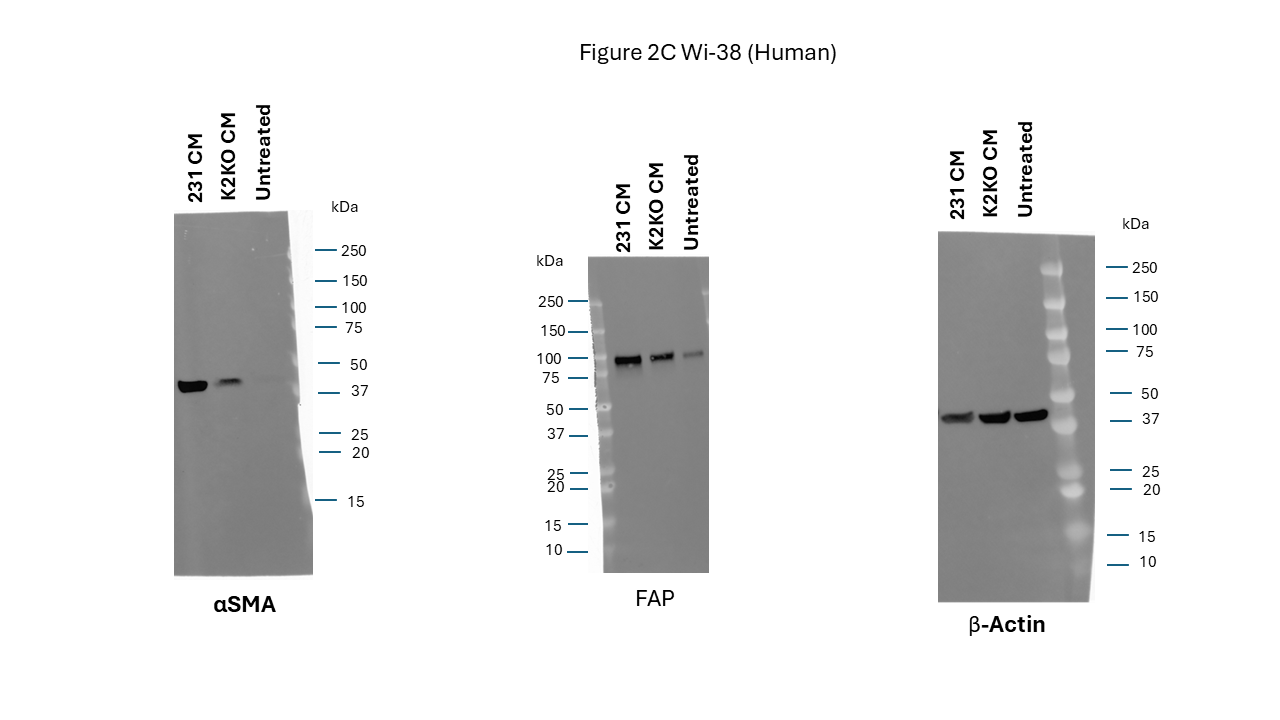

Supplement: Supplementary file 1 [file cells-14-01034-s001.zip › cells-3714860-supplementary/Uncropped WB/Uncropped WB for Figure 2C Wi-38.TIF]

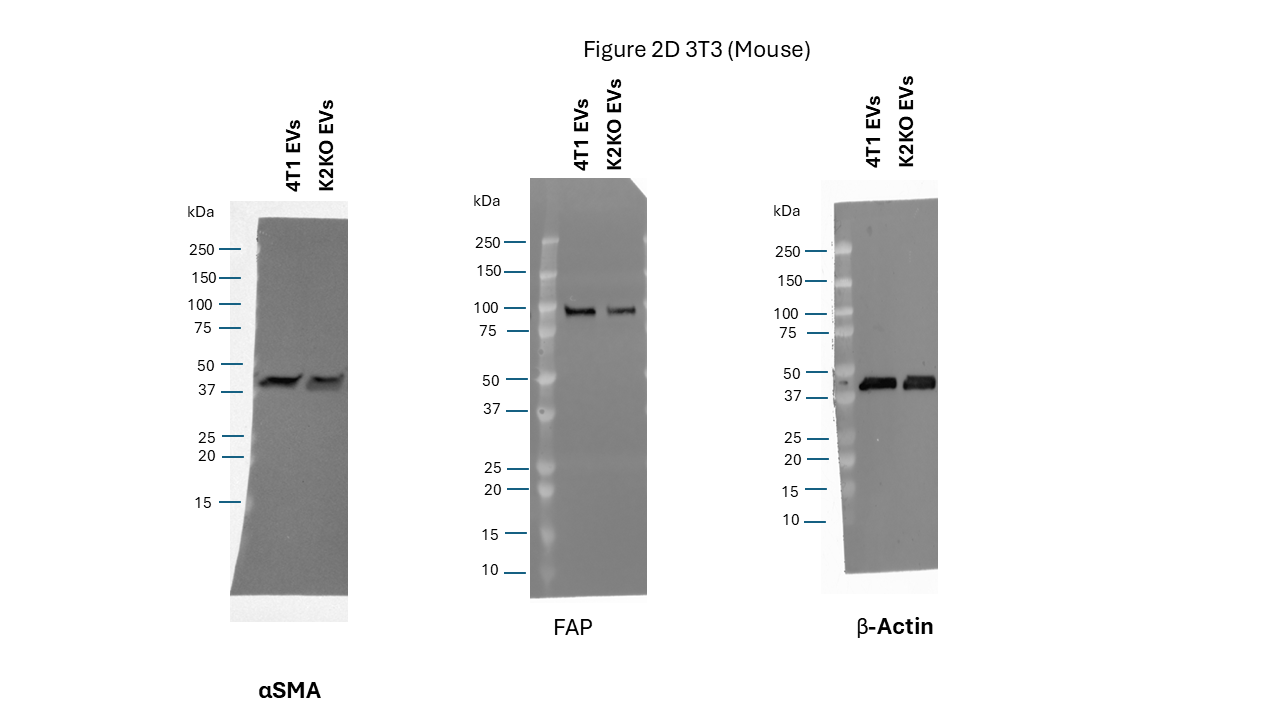

Supplement: Supplementary file 1 [file cells-14-01034-s001.zip › cells-3714860-supplementary/Uncropped WB/Uncropped WB for Figure 2D 3T3.TIF]

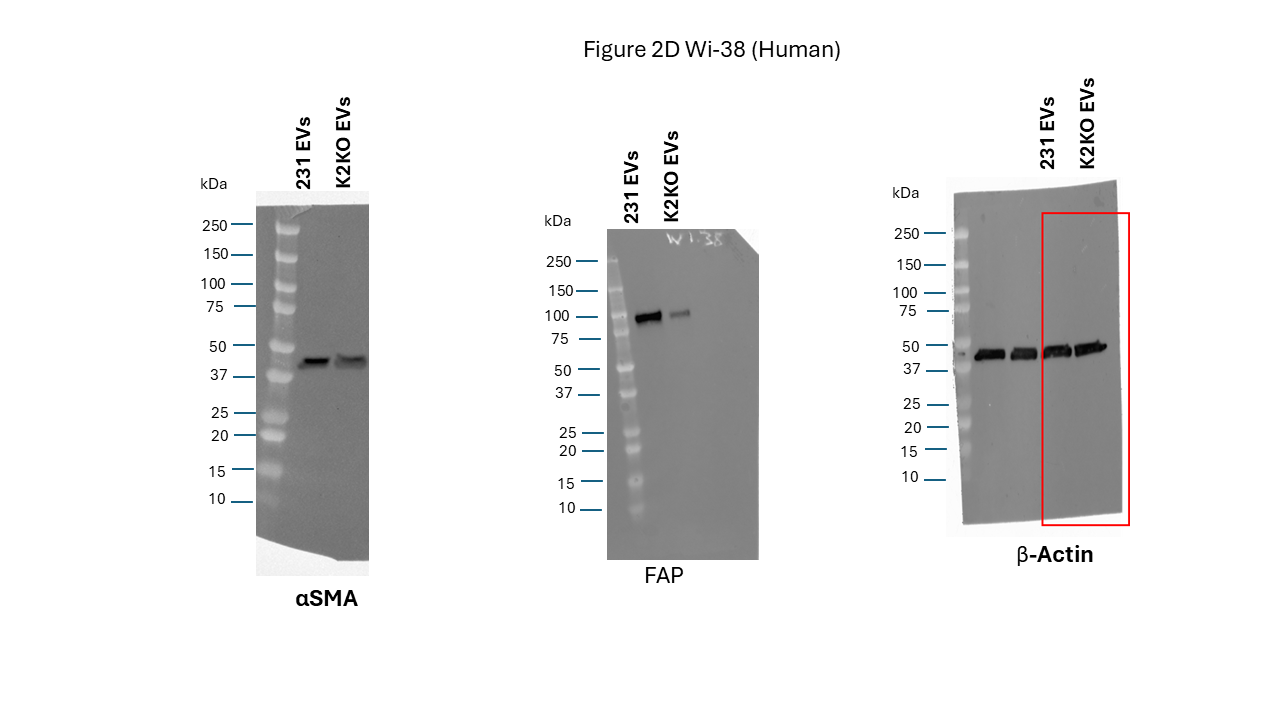

Supplement: Supplementary file 1 [file cells-14-01034-s001.zip › cells-3714860-supplementary/Uncropped WB/Uncropped WB for Figure 2D Wi-38.TIF]
